# Prevalence and Predictors of Priapism Among Patients with Sickle Cell Disease: A Cross-Sectional Study

**DOI:** 10.3390/medicina62020278

**Published:** 2026-01-28

**Authors:** Mortadah Alsalman, Jawad Alnajjar, Ridha Alhussain, Abdulah Alshakhs, Zainab Alkhmis, Abdullah Alghafli, Hassan Alradhi, Mujtaba Alzuwayr, Nawal Eltayeb Omer Mohammed, Zaenb Alsalman, Ahmed Al-Suliman

**Affiliations:** 1 Department of Medicine, College of Medicine, King Faisal University, Al-Ahsa 31982, Saudi Arabia; moalsalman@kfu.edu.sa; 2 College of Medicine, King Faisal University, Al-Ahsa 31982, Saudi Arabia; jawd.alnajjar@gmail.com (J.A.); ridhamedical@gmail.com (R.A.); abdullahafif250@gmail.com (A.A.); zainabalkhmis@gmail.com (Z.A.); amalghafli29@gmail.com (A.A.); hassa000999n@gmail.com (H.A.); mahzr33res@gmail.com (M.A.); 3 Department of Medicine, Hereditary Blood Diseases Centre, Al-Ahsa 36362, Saudi Arabia; gelas3@hotmail.com; 4 Department of Family and Community Medicine, College of Medicine, King Faisal University, Al-Ahsa 31982, Saudi Arabia; 5 Department of Medicine, AlMoosa Specialist Hospital, Al-Ahsa 36342, Saudi Arabia; ahmedms2012@hotmail.com

**Keywords:** sickle cell disease, priapism, predictors

## Abstract

*Background and Objectives*: Priapism, most commonly the ischemic (low-flow) type, is a debilitating complication affecting males with sickle cell disease (SCD), making prevention a critical aspect of care. This study aimed to determine the prevalence of priapism and identify its associated predictors among adult patients with SCD. *Materials and Methods*: This cross-sectional study was conducted at the Hereditary Blood Disorder Centre in the Eastern Province of Saudi Arabia from February 2024 to August 2025. *Results*: A total of 240 male SCD patients were included, of whom 33 (13.8%) reported a lifetime history of priapism. The median age at the first priapism episode was 29 years. Multivariate logistic regression identified a history of blood transfusion as the only significant independent predictor of priapism (aOR = 10.36, 95% CI: 1.32–81.14, *p* = 0.026). Episode frequency varied, with 33.3% of affected patients reporting episodes occurring monthly or less than weekly. Regarding timing, 45.5% of episodes occurred during sleep, while 27.3% occurred in the early morning hours. Kaplan–Meier survival curves indicated that patients receiving hydroxyurea experienced priapism at significantly younger ages compared with non-users (95% CI: 23–33 years). This difference was confirmed by a statistically significant log-rank test (*p* = 0.012), indicating a more rapid decline in the probability of remaining priapism-free with increasing age among hydroxyurea users. *Conclusions*: Priapism in SCD remains a significant clinical challenge despite available therapeutic options. Transfusion burden, alongside previously recognized predictors, serves as a key indicator of priapism risk and underscores the importance of early identification and intervention. Although hydroxyurea is effective in managing several complications of sickle cell disease, priapism was observed in patients despite its use, and episodes continued to occur with increasing age; however, these findings may be influenced by confounding factors such as age, disease severity, and transfusion history.

## 1. Introduction

Sickle cell disease (SCD) is the most prevalent hereditary blood disorder, resulting from a missense mutation in the HBB gene, which encodes the β-globin subunit of hemoglobin. It is estimated that around eight million individuals globally are affected by this condition. In Saudi Arabia, approximately 0.26% of the population is diagnosed with SCD, while about 4% carry the sickle cell trait. Notably, the Eastern Province of Saudi Arabia reports the highest prevalence of SCD, with around 1.2% of its population affected [1,2]. The condition holds significant and increasing relevance for global public health due to its association with complex healthcare needs, reduced life expectancy, chronic disabilities, and considerable expenses related to medical care [2,3].

Sickle cell disease is characterized by a detrimental cycle that involves a complex interaction of genetic factors, the polymerization of hemoglobin S (HbS), impaired biorheology, enhanced adhesion-mediated vaso-occlusion, hemolysis-induced endothelial dysfunction, and the activation of sterile inflammation. It is essential to acknowledge the substantial discrepancy between genotype and phenotype in this context [4,5]. Chronic hemolytic anemia and intermittent acute pain episodes are considered defining characteristics of SCD. However, the intricate nature of the disease’s pathophysiology results in clinical heterogeneity [6]. Consequently, patients with SCD may present with a diverse array of chronic complications, including chronic pain and end-organ damage, which can include stroke, acute kidney injury, and avascular necrosis. In addition, acute complications of SCD may manifest as vaso-occlusive crises (VOC), acute chest syndrome (ACS), and priapism [7,8].

Priapism is acknowledged as a urological emergency characterized by a painful, involuntary erection that occurs in the absence of sexual stimulation and is not alleviated by ejaculation [9,10]. This condition can endure for over four hours, particularly in cases categorized as major priapism. There are two primary forms of priapism: ischemic and non-ischemic. Non-ischemic priapism represents only 5% of cases and is considered an exceptionally rare disorder, typically resulting from perineal or penile trauma and characterized by unregulated arterial inflow. In contrast, ischemic priapism accounts for nearly 95% of cases and is the most common form seen in patients with sickle cell disease, typically presenting as painful, recurrent episodes that can lead to erectile dysfunction if not treated promptly [11,12]. Therefore, prevention of priapism is critical to avoid any serious adverse effects. Nonetheless, a significant portion of patients experiencing priapism, approximately 50%, do not pursue any treatment for their condition. Furthermore, fewer than 10% of these patients obtain appropriate medical care. This pattern is influenced by several factors, including patients’ feelings of embarrassment, the intermittent nature of priapism episodes, and a lack of knowledge and understanding regarding the condition. Consequently, many individuals are reluctant to seek medical attention due to these barriers [11,13]. This study aims to investigate the prevalence of priapism in adult patients with SCD and to identify the predictors associated with the occurrence of priapism within this patient population.

## 2. Materials and Methods

### 2.1. Study Design and Setting

This cross-sectional study was conducted at the Hereditary Blood Disorder Center in the Al-Ahsa region of Saudi Arabia between February 2024 and August 2025. The center serves as a referral hub for patients with inherited hematological disorders and was selected due to the region’s high prevalence of SCD. Our study followed the Strengthening the Reporting of Observational Studies in Epidemiology (STROBE) guidelines for cross-sectional studies to ensure structured reporting and methodological transparency.

### 2.2. Study Population and Eligibility Criteria

All male patients diagnosed with SCD who visited the center during the study period were invited to participate. Patients were categorized into two groups based on whether or not they experienced priapism at any point in their lifetime. The study aimed to include all eligible patients during the data collection period, ensuring a representative sample of the local SCD population. Inclusion criteria included male patients with a confirmed SCD diagnosis of any age who provided informed consent or assent with parental consent for minors, while exclusion criteria included female patients, patients with incomplete medical records preventing assessment of key study variables, and those who declined participation.

### 2.3. Data Collection Procedures

Data were collected through structured, face-to-face interviews using a questionnaire developed specifically for this study. The questionnaire included sections on demographic information, medical history of SCD, detailed characteristics of any priapism episodes, including age at first episode, episode duration, frequency, timing patterns, relationship to sexual activity, relationship to other SCD crises, and management strategies utilized by patients. Clinical laboratory data were extracted from medical records, including complete blood count parameters, hemoglobin electrophoresis results, and treatment history. Self-reported measurements such as height, weight, and neck circumference were obtained via follow-up phone calls when necessary. Body mass index (BMI) was calculated from height and weight measurements and categorized as underweight, normal, overweight, or obese according to standard classifications. Obstructive sleep apnea (OSA) risk was assessed using the validated STOP-Bang questionnaire [14].

### 2.4. Study Variables and Outcomes

The primary outcome was the prevalence of priapism among SCD patients, defined as any history of an unwanted penile erection lasting more than 30 min. Secondary outcomes included age at first priapism episode, predictors of priapism occurrence, predictors of severe priapism requiring hospitalization or surgical intervention, and predictors of frequent priapism, defined as episodes occurring weekly or more often. Potential predictor variables assessed included age at study enrollment, presence of comorbidities including stroke history, current use of hydroxyurea therapy, suboptimal hydroxyurea dosing, defined as 15 mg/kg/day or less, history of splenectomy, BMI, OSA risk categorized as high versus others, lifetime history of blood transfusions, total number of lifetime blood transfusions, crisis-related hospitalizations per year, emergency room visits per year, white blood cell count, hemoglobin level, platelet count, fetal hemoglobin percentage (HbF), mean corpuscular volume (MCV) with the threshold at 80 fL, hemoglobin A2 percentage (HbA2) with the threshold at 4%, lactate dehydrogenase levels, and marital status.

### 2.5. Statistical Analysis

Continuous variables were assessed for normality using the Shapiro–Wilk test and are presented as median with interquartile range (IQR) for non-normally distributed data. Categorical variables are presented as frequencies and percentages. Comparisons between patients with and without priapism history were performed utilizing the Mann–Whitney U test for continuous variables and chi-squared or Fisher’s exact test for categorical variables as appropriate. Univariate logistic regression was conducted to identify potential predictors of priapism, with results presented as odds ratios (ORs) with 95% confidence intervals (CIs) and *p*-values. Variables with *p*-value < 0.20 in univariate analysis were considered for inclusion in multivariate logistic regression models. However, due to the limited number of events relative to the number of potential predictors, multivariate models were constructed with careful consideration of events-per-variable ratios to avoid overfitting.

Receiver operating characteristic (ROC) curve analysis was performed to determine the optimal cut-off platelet count threshold for predicting priapism risk, with sensitivity, specificity, and area under the curve (AUC) calculated. Age-stratified subgroups were conducted to evaluate potential effect modification by age on the relationship between hydroxyurea use and priapism occurrence. Kaplan–Meier survival analysis was utilized to estimate the probability of remaining priapism-free by age, comparing patients who used hydroxyurea versus those who did not, with the log-rank test utilized to assess differences between the groups. For the survival analysis, the event was defined as the first priapism episode, the time variable was age at first episode for those with priapism or current age for those without priapism, and censoring occurred at current age for patients who never experienced priapism. Missing data for episode frequency and other descriptive priapism characteristics were not imputed and were excluded from descriptive analyses, whereas median imputation was applied only to continuous variables included in regression models. All statistical tests were two-tailed, with *p*-value < 0.05 considered statistically significant. Statistical analyses were performed utilizing RStudio statistical software with R version 4.4.2.

## 3. Results

### 3.1. Baseline Characteristics and Prevalence of Priapism

**Figure 1:**
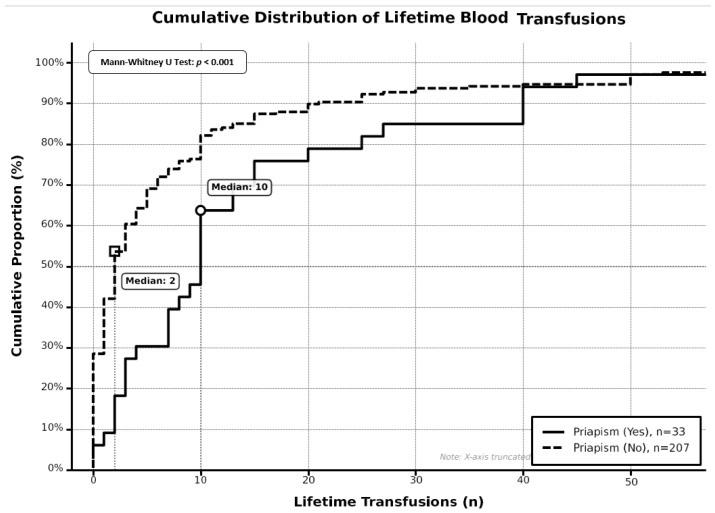
Cumulative distribution of lifetime blood transfusions by priapism status.

**Figure 2:**
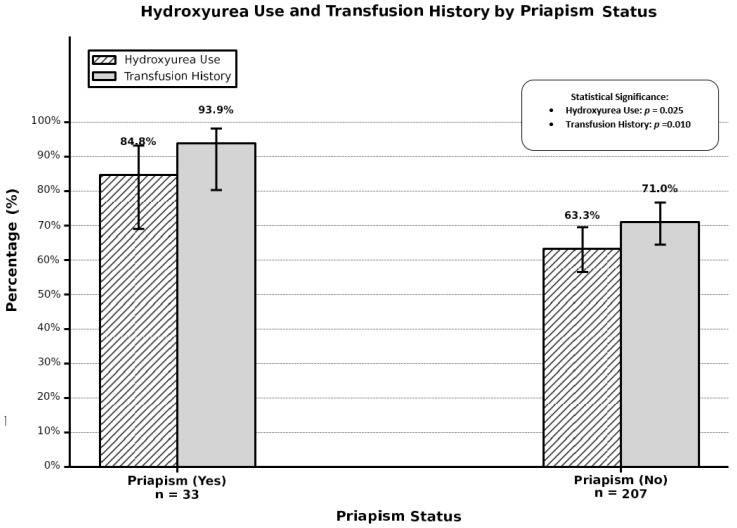
Hydroxyurea use and transfusion history according to priapism status.

**Figure 3:**
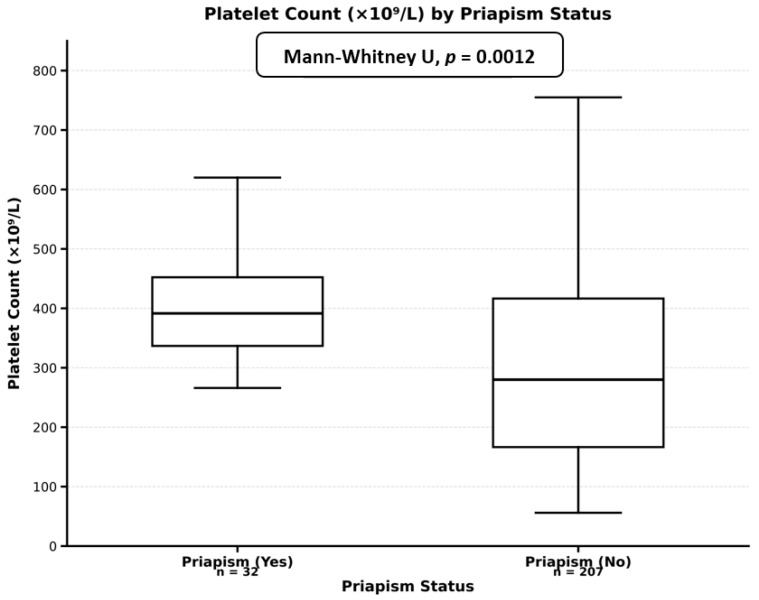
Platelet counts according to priapism status.

A total of 240 male SCD patients were included in our study, with 33 patients (13.8%) reporting a history of priapism at any point in their lifetime and 207 patients (86.3%) reporting no history of priapism. Baseline characteristics stratified by priapism history are presented in Table 1. Patients with a priapism history demonstrated a higher median age compared to those without priapism history; however, this difference did not reach statistical significance (37.0 years versus 32.0 years, *p*-value = 0.080). BMI showed no significant difference between groups (23.1 kg/m^2^ versus 23.7 kg/m^2^, *p*-value = 0.382). Annual crisis hospitalizations were similar between groups (median one hospitalization per year in both groups, *p*-value = 0.327); however, patients with a priapism history showed marginally higher emergency room visits per year (5.0 versus 4.0 visits, *p*-value = 0.090). Lifetime blood transfusion history differed significantly between groups, with patients with a priapism history having received significantly more transfusions (median of ten transfusions versus two transfusions, *p*-value < 0.001) (Figure 1). Transfusion history was present in 93.9% of patients with priapism compared to 71.0% of patients without priapism (*p*-value = 0.005) (Figure 2). Laboratory parameters revealed significant differences in platelet counts, with patients with priapism demonstrating higher median platelet counts (391.5 × 10^9^/L versus 280.0 × 10^9^/L, *p*-value = 0.001) (Figure 3). Mean corpuscular volume was significantly higher in patients with a priapism history (87.8 fL versus 77.9 fL, *p*-value = 0.025). Hydroxyurea use was more prevalent in patients with a priapism history (84.8% versus 63.3%, *p*-value = 0.015) (Figure 2). White blood cell count, hemoglobin level, HbA2, and HbF showed no significant differences between groups. Marital status demonstrated a trend toward significance, with a higher proportion of married patients in the priapism group (72.7% versus 53.6%, *p*-value = 0.065). OSA risk and smoking status showed no significant associations with priapism history.

### 3.2. Characteristics of Priapism Episodes

The episode characteristics of the 33 patients with a priapism history are detailed in Table 2. The median age at the first priapism episode was 29 years (IQR: 21–35 years), with a range of 3 to 55 years. Typical episode duration was 60 min (IQR: 30–180 min), ranging up to 180 min. Episode frequency varied, with 27.3% of the patients reporting infrequent episodes occurring less than monthly, 33.3% reporting monthly to less than weekly episodes, 12.1% reporting weekly to less than daily episodes, 6.1% reporting daily or more frequent episodes, and 21.2% with missing frequency data. Regarding timing patterns, 45.5% of episodes occurred during sleep, 27.3% occurred in the early morning hours, 18.2% had no specific timing pattern, and 9.1% had missing timing data. Relationship to sexual activity showed that 36.4% of episodes were not associated with sexual intercourse, 30.3% were related to sexual activity, 12.1% occurred during sexual intercourse specifically, and 21.2% had missing data. We found that 72.7% of priapism episodes occurred while patients were receiving hydroxyurea therapy, and 60.6% of episodes were unrelated to other SCD crises. Only 6.1% of patients underwent surgical intervention to prevent recurrent priapism episodes.

### 3.3. Univariate and Multivariate Predictors of Priapism

**Figure 4:**
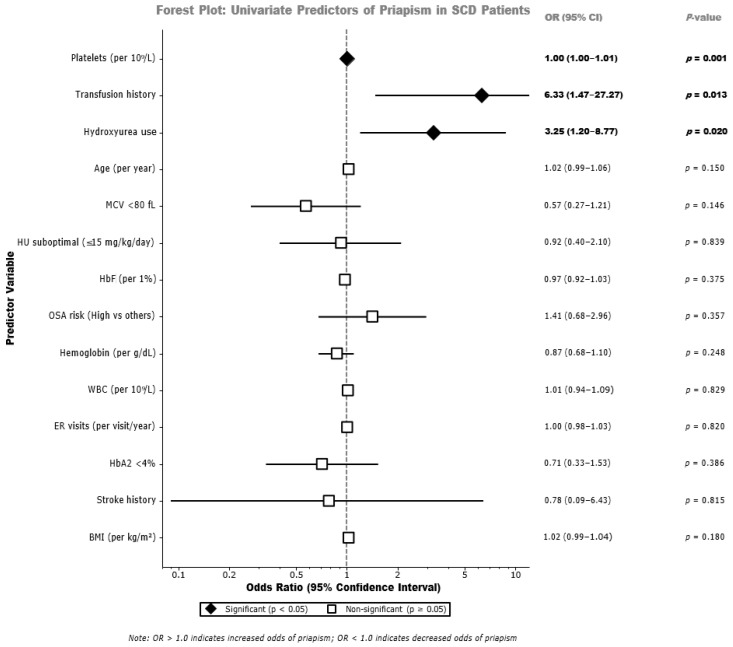
Forest plot of univariate predictors of priapism in sickle cell disease.

Univariate logistic regression identified several potential predictors of priapism occurrence, as shown in Table 3 and visualized in Figure 4. Transfusion history demonstrated a significant association with priapism (OR = 6.33, 95% CI: 1.47–27.27, *p*-value = 0.013). Hydroxyurea use demonstrated a significant association with priapism history (OR = 3.25, 95% CI: 1.20–8.77, *p*-value = 0.020). Platelet count per 10^9^/L increment was significantly associated with increased priapism risk (OR = 1.00, 95% CI: 1.00–1.01, *p*-value = 0.001). Age per year showed a marginal association (OR = 1.02, 95% CI: 0.99–1.06, *p*-value = 0.150). Emergency room visits per year showed no significant association (OR = 1.00, 95% CI: 0.98–1.03, *p*-value = 0.820). Other variables, including BMI, stroke history, HbA2 less than 4%, white blood cell count, hemoglobin level, OSA risk, HbF percentage, suboptimal hydroxyurea dosing, and MCV less than 80 fL, did not demonstrate significant associations in univariate analysis. Multivariate logistic regression integrating age, suboptimal hydroxyurea dosing, MCV less than 80 fL, OSA risk, emergency room visits, and transfusion history as covariates identified transfusion history as the only significant independent predictor of priapism (aOR = 10.36, 95% CI: 1.32–81.14, *p*-value = 0.026). Age showed an aOR of 1.02 (95% CI: 0.98–1.06, *p*-value = 0.319), suboptimal hydroxyurea dosing showed an aOR of 1.01 (95% CI: 0.42–2.43, *p*-value = 0.983), MCV less than 80 fL showed an aOR of 0.58 (95% CI: 0.23–1.44, *p*-value = 0.240), OSA risk showed an aOR of 0.84 (95% CI: 0.33–2.14, *p*-value = 0.719), and emergency room visits showed an aOR of 1.00 (95% CI: 0.98–1.03, *p*-value = 0.698), Table 3.

### 3.4. Hematological Parameters Among Priapism Patients by Hydroxyurea Status

Among patients with a priapism history, subgroup analysis comparing hematological parameters between those receiving hydroxyurea therapy versus those not receiving hydroxyurea revealed no statistically significant differences across all three parameters investigated. Mean hemoglobin levels were comparable between the hydroxyurea group (10.09 ± 1.70 g/dL, n = 14) and the non-hydroxyurea group (9.80 ± 1.11 g/dL, n = 3), with a mean difference of +0.29 g/dL (*p*-value = 0.705, Cohen’s d = 0.18, negligible effect size). Similarly, fetal hemoglobin levels showed no significant difference between the hydroxyurea group (16.78 ± 7.57%, n = 25) and the non-hydroxyurea group (13.84 ± 7.58%, n = 5), with a mean difference of +2.94% (*p* = 0.290, Cohen’s d = 0.39, small-to-medium effect size). Platelet counts were also comparable between the hydroxyurea group (462.43 ± 317.26 × 10^9^/L, n = 28) and the non-hydroxyurea group (343.80 ± 153.09 × 10^9^/L, n = 5), with a mean difference of +118.63 × 10^9^/L (*p*-value = 0.564, Cohen’s d = 0.39, small-to-medium effect size). While all three parameters demonstrated numerically higher values in the hydroxyurea group, none of these differences reached statistical significance, likely attributable to the limited sample size in the non-hydroxyurea subgroup.

### 3.5. Predictors of Severe and Frequent Priapism Among Affected Patients

Among the 33 patients with a priapism history, analyses were conducted to identify predictors of severe priapism requiring hospitalization or intervention and frequent priapism occurring weekly or more often (Table 4). For severe priapism, univariate analysis showed no significant predictors, with suboptimal hydroxyurea dosing (OR = 0.33, 95% CI: 0.07–1.60, *p*-value = 0.169), high OSA risk (OR = 0.38, 95% CI: 0.04–3.65, *p*-value = 0.398), age per year (OR = 0.98, 95% CI: 0.90–1.07, *p*-value = 0.695), and HbF per 1% (OR = 0.95, 95% CI: 0.84–1.07, *p*-value = 0.373) all showing non-significant associations. A multivariate model for severe priapism, including suboptimal hydroxyurea dosing and high OSA risk, also demonstrated no significant predictors.

For frequent priapism, a univariate model in a similar manner showed no significant associations, with suboptimal hydroxyurea dosing (OR = 1.10, 95% CI: 0.27–4.55, *p*-value = 0.895), high OSA risk (OR = 2.92, 95% CI: 0.48–17.86, *p*-value = 0.247), age per year (OR = 0.99, 95% CI: 0.92–1.06, *p*-value = 0.712), and HbF per 1% (OR = 0.96, 95% CI: 0.87–1.06, *p*-value = 0.405) all non-significant. The limited sample size and low number of events constrained the statistical power for these subgroup analyses.

### 3.6. Platelet Count Threshold Analysis for Priapism Risk

**Figure 5:**
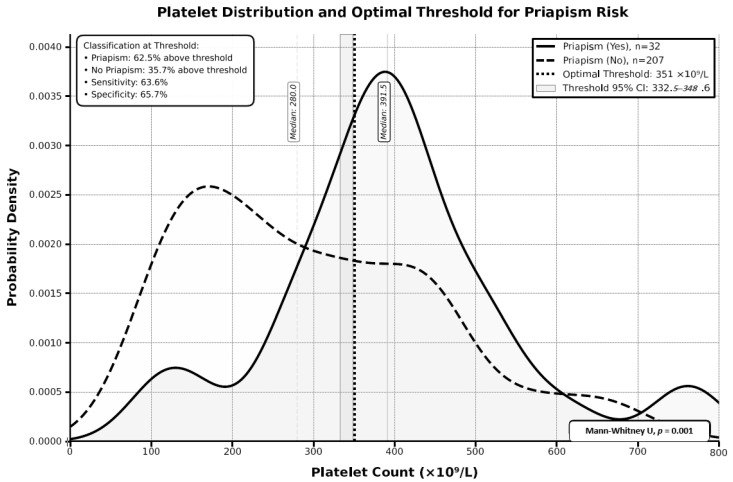
Platelet distribution and optimal threshold for priapism risk.

ROC curve assessment was performed to determine the optimal platelet count threshold for predicting priapism risk (Table 5). The optimal threshold was identified at 351 × 10^9^/L (95% CI: 332.5–348.6 × 10^9^/L) using Youden’s index method. At this threshold, sensitivity was 63.6%, and specificity was 65.7%. Classification performance showed that 62.5% of patients with priapism had platelet counts above the threshold, while 35.7% of patients without priapism had counts above the threshold. The Mann–Whitney U test confirmed the significant difference in platelet distributions between groups (median 391.5 versus 280.0 × 10^9^/L, *p*-value = 0.001). The density distributions demonstrated distinct separation between the two groups, with patients with priapism showing higher platelet counts overall (Figure 5).

### 3.7. Age-Stratified Analysis of the Hydroxyurea Effect on Priapism

Age-stratified subgrouping was conducted to evaluate potential effect modification by age on the relationship between hydroxyurea use and priapism occurrence (Table 6). In patients aged less than 30 years, hydroxyurea use showed no significant association with priapism (OR = 0.77, 95% CI: 0.13–4.47, *p*-value = 0.768), with 90.9% of priapism patients and 72.3% of non-priapism patients in this age group using hydroxyurea, as shown in Figure 2. In contrast, among patients aged 30 years or older, hydroxyurea use demonstrated a strong, significant association with priapism occurrence (OR = 5.97, 95% CI: 1.71–20.88, *p*-value = 0.005), with 81.8% of priapism patients and 53.4% of non-priapism patients in this age group using hydroxyurea. The overall effect across both age strata resulted in OR = 3.25 (95% CI: 1.20–8.77, *p*-value = 0.020), consistent with the univariate model. Interaction analysis revealed a significant age-by-hydroxyurea interaction (interaction OR = 7.78, 95% CI: 1.01–59.81, *p*-value = 0.049), indicating that the association between hydroxyurea use and priapism risk differs significantly between younger and older patients.

### 3.8. Survival Analysis of Age at First Priapism Episode by Hydroxyurea Use

**Figure 6:**
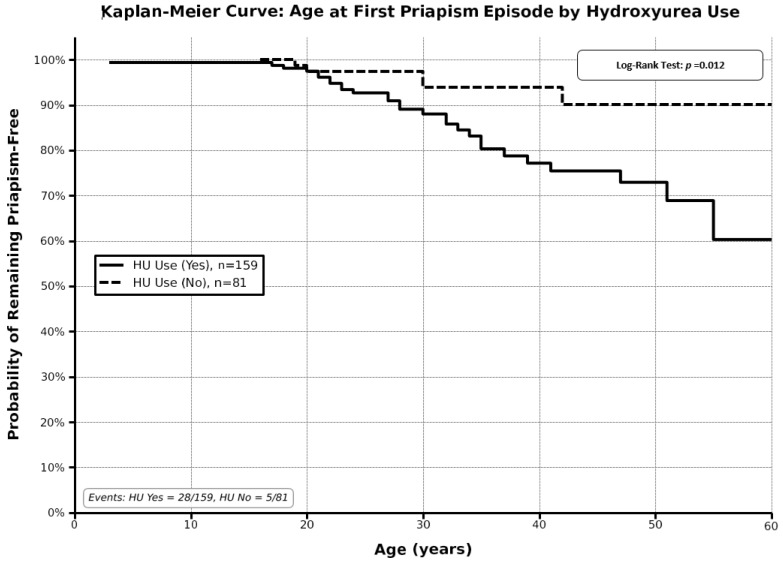
Kaplan–Meier curve of age at first priapism episode by hydroxyurea use.

Kaplan–Meier survival analysis was performed to evaluate age at first priapism episode stratified by hydroxyurea use status (Table 7). The analysis included a total of 240 patients, with 159 patients who used hydroxyurea and 81 patients who did not use hydroxyurea. Among hydroxyurea users, 28 events (first priapism episodes) occurred during follow-up, while five events occurred among non-users. The survival curves demonstrated that hydroxyurea users experienced priapism at significantly younger ages compared to non-users. The median age at the first priapism episode was 28 years (95% CI: 23–33 years) for hydroxyurea users, while the median was not reached in the non-user group, with the survival curve suggesting a later onset. Log-rank test confirmed a statistically significant difference between the two survival curves (*p*-value = 0.012), indicating that hydroxyurea use is associated with earlier age at priapism onset. The probability of remaining priapism-free decreased more rapidly with age in the hydroxyurea user group, with around 75% remaining priapism-free by age 40 years, compared to around 90% in the non-user group at the same age (Figure 6).

## 4. Discussion

The etiology of priapism has been documented over an extensive period; however, accurately determining its prevalence remains a challenge, with an estimated incidence of 1.1 to 1.5 cases per 100,000 person-years. The common etiologies of priapism can vary, encompassing both hematological abnormalities and structural deformities [15]. Priapism is a serious complication of sickle cell disease (SCD) in males that often goes underreported and underrecognized. Approximately 33% of adult men diagnosed with SCD are affected by priapism [12]. Within Saudi Arabia, research addressing the prevalence and characteristics of priapism among sickle cell patients is limited. Reported prevalence rates vary significantly, ranging from 0% to 17%, with most studies focusing on the Eastern Province of Saudi Arabia [16,17]. In our study, we identified that approximately 14% of patients diagnosed with sickle cell disease experience priapism at least once during their lifetime, with the median age of occurrence being 29 years. Notably, episodes can manifest as early as three years of age. This prevalence is significantly lower than the global average but aligns closely with previously reported local studies. This finding may be partially attributed to the higher average levels of hemoglobin F in our study region, where Arab Indian haplotypes are more prevalent [17,18]. Notably, the maximum duration of priapism recorded among our study population was approximately three hours, which did not fulfill the criteria for what is classified as major priapism [12,19]. Nevertheless, nearly half of the participants reported experiencing episodes of priapism less frequently than once a month, without any discernible indicators of increased frequency or severity, such as episodes necessitating hospitalization or other medical and surgical interventions.

The process of penile erection is a complex physiological phenomenon that involves a series of neurological, vascular, and hormonal interactions. This process is triggered by various stimuli, such as auditory, visual, and olfactory cues, as well as localized factors that influence the penis. A critical mediator in this process is nitric oxide (NO), which is essential for facilitating erections. The release of NO from vascular endothelial cells and penile nerve terminals is fundamental to the establishment of an erection [20,21]. Research indicates that testosterone regulates the production of nitric oxide and enhances endothelial function. Furthermore, it is important to acknowledge that testosterone levels exhibit diurnal variation, typically peaking in the morning and subsequently declining throughout the day [22,23]. In alignment with the previously mentioned findings, we discovered that approximately one-third of participants experience priapism in the early morning, while the majority report nocturnal episodes. We hypothesized that nocturnal hypoxia may contribute to the increased frequency of events occurring at night, leading to red blood cell sickling and vaso-occlusion in the penile microvasculature. However, neither obesity nor a higher risk of obstructive sleep apnea, which is associated with lower oxygen levels, demonstrated a significant correlation with the occurrence of priapism [24,25]. Moreover, a comparable number of priapism incidents were recorded both in the presence and absence of sexual activity. Nonetheless, there was a discernible trend indicating that married individuals were more likely to experience priapism. Furthermore, more than two-thirds of the episodes occurred independently, without the coexistence of other medical crises, and irrespective of any prior history of hospitalization or acute chest syndrome.

Several treatment options have been established and have demonstrated their effectiveness in managing patients with acute and chronic complications related to SCD. Nevertheless, there remains a lack of definitive evidence regarding the optimal treatment strategy to prevent the recurrence of priapism in patients with SCD [24,26]. For example, the efficacy of transfusion in alleviating established acute priapism has not been substantiated by randomized controlled trials. In contrast, allogeneic hematopoietic stem cell transplantation (HSCT) is showing promising outcomes, not only in alleviating common SCD-related morbidities, such as vaso-occlusive pain, but also potentially in reducing the incidence of SCD-related priapism [27,28]. Among all therapeutic alternatives, hydroxyurea exhibits considerable benefits for individuals with SCD experiencing priapism, attributable to its multiple mechanisms of action, including nitric oxide (NO) donation, decreased hemolysis, and elevated levels of fetal hemoglobin (HbF) [19,29]. Conversely, findings from our study indicate that hydroxyurea users experienced episodes of priapism at significantly younger ages, and their probability of remaining free from priapism diminished more rapidly with advancing age compared to non-users. These results are consistent with the emerging body of evidence indicating an increased incidence of priapism with advancing age, although the underlying pathophysiological mechanisms remain insufficiently understood. Importantly, this association should be interpreted cautiously, as hydroxyurea use likely reflects greater disease severity and transfusion burden rather than a direct treatment effect.

Recurrent ischemic priapism is a prevalent and debilitating complication affecting males with SCD. Consequently, the prevention of priapism is vital to avert prolonged complications, achieved through the identification of factors that may predict its incidence and recurrence. Previous studies indicate that advancing age and a higher frequency of priapism episodes are significant predictors of priapism occurrence among patients with SCD. Males with SCD who have experienced at least three priapism episodes in the preceding 12 months are at considerable risk for recurrent priapism within the subsequent three months. Furthermore, minor episodes of priapism are associated with an increased likelihood of at least one major episode [11,30]. In our investigation, individuals with priapism exhibited a greater transfusion burden compared to those without priapism, and this was a significant predictor of priapism. However, it is difficult to discriminate whether the transfusion burden increased as a preventive strategy or due to an increased rate of hemolysis in these patient populations. From a laboratory perspective, higher platelet counts, particularly values exceeding 390 × 10^9^/L, were associated with priapism risk; however, thrombocytopenia in SCD is multifactorial and may also be influenced by factors such as splenic sequestration, including hypersplenism, which has been described in patients with persistent fetal hemoglobin and the Arab Indian haplotype. Additionally, other complete blood count (CBC) parameters and hemoglobin F levels did not serve as reliable predictors either.

This study elucidates that priapism in our region, characterized by Arab Indian haplotypes with elevated mean hemoglobin F levels, is less prevalent and typically manifests as a non-major form when compared to global data. Additionally, patients experiencing priapism demonstrate a higher transfusion burden, which may reflect an increased incidence of hemolysis within this patient population. Despite a lack of strong correlation between an increased risk of obstructive sleep apnea (OSA) and priapism, nocturnal priapism was found to be more prevalent than morning priapism, regardless of sexual arousal. This study is constrained by the relatively small number of priapism events, which limits statistical power and generalizability, and requires restricting multivariate regression to minimize overfitting. In addition, the retrospective, questionnaire-based design may be subject to recall and reporting bias, particularly for a sensitive outcome such as priapism. Furthermore, there is an absence of documented duration of hydroxyurea treatment, which would help clarify whether the accumulated dosage of hydroxyurea is effective in reducing the frequency of priapism occurrences. Future research is essential to investigate the relationship between nocturnal hypoxemia and the incidence of priapism. Moreover, it is imperative to assess testosterone deficiency in these patients, as testosterone plays a critical role in regulating nitric oxide release and enhancing endothelial function. It is anticipated that testosterone levels decrease with advancing age, potentially accounting for the increased incidence of priapism observed in older individuals despite hydroxyurea treatment. Additionally, evaluating haptoglobin levels in individuals with priapism compared to those who have not experienced the condition could yield valuable predictive insights and inform the selection of targeted therapeutic interventions.

## 5. Conclusions

In conclusion, priapism in sickle cell disease (SCD) presents considerable clinical challenges, even with existing therapeutic options, thus revealing a significant gap in the current treatment paradigms. A constellation of contemporary predictors, including transfusion burden along with previously validated predictors, serves as a reliable indicator for the occurrence of priapism and underscores the necessity for timely early interventions. Although hydroxyurea is known to effectively manage various complications associated with SCD, it does not delay the initial episode of priapism, and patients continue to experience these events as they progress in age. Moreover, the increased burden of transfusion in patients experiencing priapism indicates a higher rate of hemolysis, which may represent a potential target for future treatment strategies in this patient population.

## Figures and Tables

**Table 1:** Baseline characteristics by priapism history.

Variable	Priapism (Yes) n = 33	Priapism (No) n = 207	*p*-Value
**Demographics:**			
Age (years)	37.0 (31.0–43.0)	32.0 (25.0–43.0)	0.080
BMI (kg/m^2^)	23.1 (20.2–24.9)	23.7 (20.2–26.9)	0.382
**Marital status:**			
Married	24 (72.7%)	111 (53.6%)	0.065
Single	8 (24.2%)	94 (45.4%)	
Other	1 (3.0%)	2 (1.0%)	
**Healthcare Utilization:**			
Crisis hospitalizations per year	1.0 (0.0–3.0)	1.0 (0.0–2.0)	0.327
ER visits per year	5.0 (4.0–12.0)	4.0 (2.0–10.0)	0.090
**Transfusion History:**			
Any transfusion history	31 (93.9)	147 (71.0)	0.005
Lifetime transfusions (n)	10.0 (3.0–15.0)	2.0 (0.0–8.0)	<0.001
**Hematologic Parameters:**			
WBC (×10^9^/L)	10.7 (6.9–13.7)	8.8 (5.6–12.8)	0.323
Hemoglobin (g/dL)	11.0 (9.8–11.5)	10.7 (9.3–13.0)	0.942
Platelets (×10^9^/L)	391.5 (336.8–452.2)	280.0 (166.5–416.5)	0.001
MCV (fL)	87.8 (72.3–96.5)	77.9 (69.5–87.3)	0.025
MCV < 80 fL	13 (39.4)	110 (53.1)	0.142
HbA2 (%)	3.0 (2.6–3.6)	3.0 (2.7–3.8)	0.919
HbA2 < 4%	12 (36.4)	92 (44.4)	1.000
HbF (%)	13.8 (11.7–21.9)	17.1 (13.6–23.1)	0.168
**Treatment and Comorbidities:**			
Hydroxyurea use	28 (84.8%)	131 (63.3%)	0.015
HU suboptimal (≤15 mg/kg/day)	16 (48.5%)	77 (37.2%)	0.839
Current smoker	14 (42.4%)	55 (26.6%)	0.062
Stroke history	1 (3.0%)	8 (3.9%)	1.000
High OSA risk	18 (54.5%)	95 (45.9%)	0.184

Notes: Data presented as median (IQR) for continuous variables and n (%) for categorical variables. Continuous variables compared using the Mann–Whitney U test; categorical variables compared using chi-squared test or Fisher’s exact test as appropriate. Abbreviations: BMI, body mass index; ER, emergency room; WBC, white blood cell count; MCV, mean corpuscular volume; HbA2, hemoglobin A2; HbF, fetal hemoglobin; HU, hydroxyurea; OSA, obstructive sleep apnea; IQR, interquartile range.

**Table 2:** Priapism characteristics among patients with priapism.

Characteristic	Summary (n = 33)
**Episode Characteristics:**	
Age at first priapism episode (years)	29.0 (21.8–35.0)
Typical episode duration (minutes)	60.0 (30.0–180.0)
Episodes per year	12.0 (1.0–12.0)
**Episode Frequency Category:**	
Infrequent (<monthly)	9 (27.3%)
Monthly to <weekly	11 (33.3%)
Weekly to <daily	4 (12.1%)
Daily or more	2 (6.1%)
Missing data	7 (21.2%)
**Common Time of Priapism:**	
During sleep (night)	15 (45.5%)
Early morning	9 (27.3%)
Sexual interaction	3 (9.1%)
Other	6 (18.2%)
**Timing Relative to Sexual Intercourse:**	
Does not occur with sexual intercourse	12 (36.4%)
Unrelated	6 (18.2%)
Post-intercourse	4 (12.1%)
Pre-intercourse	3 (9.1%)
Unknown	7 (21.2%)
Not applicable	1 (3.0%)
**Treatment Context:**	
Priapism occurred while on hydroxyurea	24 (72.7%)
Priapism occurred during other SCD crises	13 (39.4%)
Surgery to prevent priapism	2 (6.1%)

Notes: Data are presented as median (IQR) for continuous variables and n (%) for categorical variables. Summaries restricted to participants with any history of priapism. Abbreviations: IQR, interquartile range; SCD, sickle cell disease.

**Table 3:** Predictors of priapism using univariate and multivariate logistic regression.

Predictor	Univariate OR (95% CI)	Univariate *p*-Value	Multivariate aOR (95% CI)	Multivariate *p*-Value
Age (per year)	1.02 (0.99–1.06)	0.150	1.02 (0.98–1.06)	0.319
BMI (per kg/m^2^)	1.02 (0.99–1.04)	0.180	-	-
ER visits (per visit/year)	1.00 (0.98–1.03)	0.820	1.00 (0.98–1.03)	0.698
Transfusion history	6.33 (1.47–27.27)	0.013	10.36 (1.32–81.14)	0.026
Hydroxyurea use	3.25 (1.20–8.77)	0.020	-	-
HU suboptimal (≤15 mg/kg/day)	0.92 (0.40–2.10)	0.839	1.01 (0.42–2.43)	0.983
Stroke history	0.78 (0.09–6.43)	0.815	-	-
MCV < 80 fL	0.57 (0.27–1.21)	0.146	0.58 (0.23–1.44)	0.240
HbA2 < 4%	0.71 (0.33–1.53)	0.386	-	-
HbF (per 1%)	0.97 (0.92–1.03)	0.375	-	-
WBC (per 10^9^/L)	1.01 (0.94–1.09)	0.829	-	-
Hemoglobin (per g/dL)	0.87 (0.68–1.10)	0.248	-	-
Platelets (per 10^9^/L)	1.00 (1.00–1.01)	0.001	-	-
OSA risk (High vs. others)	1.41 (0.68–2.96)	0.357	0.84 (0.33–2.14)	0.719

Abbreviations: OR, odds ratio; aOR, adjusted odds ratio; CI, confidence interval; BMI, body mass index; ER, emergency room; HU, hydroxyurea; MCV, mean corpuscular volume; HbA2, hemoglobin A2; HbF, fetal hemoglobin; WBC, white blood cell count; OSA, obstructive sleep apnea.

**Table 4:** Predictors of severe and frequent priapism among patients with priapism history.

Predictor.	Severe Priapism Univariate OR (95% CI)	Severe Priapism Univariate *p*-Value	Severe Priapism Multivariate aOR (95% CI)	Severe Priapism Multivariate *p*-Value	Frequent Priapism Univariate OR (95% CI)	Frequent Priapism Univariate *p*-Value
HU suboptimal (≤15 mg/kg/day)	0.33 (0.07–1.60)	0.169	0.38 (0.07–2.13)	0.272	1.10 (0.27–4.55)	0.895
OSA risk (High vs. others)	0.38 (0.04–3.65)	0.398	0.61 (0.05–7.24)	0.696	2.92 (0.48–17.86)	0.247
Age (per year)	0.98 (0.90–1.07)	0.695	-	-	0.99 (0.92–1.06)	0.712
HbF (per 1%)	0.95 (0.84–1.07)	0.373	-	-	0.96 (0.87–1.06)	0.405

Abbreviations: OR, odds ratio; aOR, adjusted odds ratio; CI, confidence interval; HU, hydroxyurea; OSA, obstructive sleep apnea; HbF, fetal hemoglobin.

**Table 5:** Detailed sensitivity analyses for significance assessment.

Analysis	Parameter	Estimate	95% CI/Interpretation
**E-Value Analysis:**			
IPTW risk ratio (HU vs. No HU)	RR	1.905	-
E-value for point estimate	E-value	3.22	Minimum RR of unmeasured confounder with both exposure and outcome to nullify observed association
E-value for CI lower bound	E-value	1.54	Minimum RR to shift CI to include null
**Multiple Imputation (m = 20):**			
Hydroxyurea use	Pooled OR	3.00	1.05–8.56
Transfusion history	Pooled OR	6.12	1.23–30.42
Platelets (per 10^9^/L)	Pooled OR	1.003	1.001–1.005
Comparison to primary analysis	HU OR difference	−0.25	Primary OR = 3.25; MI OR = 3.00 (7.7% attenuation)
**Negative Control Analysis:**			
Exposure: Hydroxyurea use			
Outcome: HbA2 < 4% (negative control)	Adjusted OR	0.86	0.02–45.19
*p*-value	*p*	0.941	No spurious association detected
Model adjustment	Covariates	—	Age, HbF, WBC, Hgb, MCV

Abbreviations: HU, hydroxyurea; OR, odds ratio; CI, confidence interval; RR, risk ratio; IPTW, inverse probability of treatment weighting; HbA2, hemoglobin A2; HbF, fetal hemoglobin; WBC, white blood cell count; Hgb, hemoglobin; MCV, mean corpuscular volume.

**Table 6:** Platelet count threshold analysis and age-stratified effects.

Analysis Component	Parameter	Value	95% CI/Range
**Threshold Breakpoint Analysis:**			
Optimal platelet threshold	Breakpoint	351.0 × 10^9^/L	-
Bootstrap mean threshold	Mean	340.6 × 10^9^/L	332.5–348.6
Bootstrap replications	B	500	-
Model selection criterion	Best AIC	Grid-searched across 10th–90th percentiles	-
**Performance at Threshold:**			
Patients above threshold (priapism group)	n (%)	21 (63.6%)	-
Patients above threshold (no priapism group)	n (%)	71 (34.3%)	-
Sensitivity	%	63.6	-
Specificity	%	65.7	-
**Clinical Context:**			
Median platelets (priapism group)	Median	391.5 × 10^9^/L	IQR: 336.8–452.2
Median platelets (no priapism group)	Median	280.0 × 10^9^/L	IQR: 166.5–416.5
Difference	Δ	111.5 × 10^9^/L	*p* = 0.001
**Age-Stratified HU Effects:**			
Age < 30 years	HU OR	0.77	0.13–4.47
Age < 30 years	*p*-value	0.768	Non-significant
Age < 30 years	Sample size	n = 89	-
Age ≥ 30 years	HU OR	5.97	1.71–20.88
Age ≥ 30 years	*p*-value	0.005	Significant
Age ≥ 30 years	Sample size	n = 151	—
Effect modification	Interaction	Present	Age modifies the HU–priapism association

Abbreviations: AIC, Akaike Information Criterion; HU, hydroxyurea; OR, odds ratio; CI, confidence interval; IQR, interquartile range.

**Table 7:** Survival analysis: age at first priapism episode by hydroxyurea use.

Analysis Component	Group/Metric	Value	95% CI/Details
**Overall Cohort:**			
Total patients	Number	240	-
Priapism events		33	13.8% cumulative incidence
Censored observations		207	86.3% priapism-free
**Hydroxyurea Use Group:**			
Sample size	Number	159	-
Priapism events		28	17.6% cumulative incidence
Censored		131	-
Median age at first priapism	Years	Not reached	>55 years
**No Hydroxyurea Group:**			
Sample size	Number	81	-
Priapism events		5	6.2% cumulative incidence
Censored		76	-
Median age at first priapism	Years	Not reached	>55 years
**Log-Rank Test:**			
Chi-square statistic	χ^2^	6.329	1 df
*p*-value	*p*	0.012	Significant at α = 0.05
**Interpretation:**			
Effect direction	-	HU users experience priapism at earlier ages	Curves diverge significantly
Clinical significance	-	17.6% vs. 6.2% cumulative incidence	2.8-fold difference
Event rate ratio	Ratio	2.84	HU vs. No HU

Abbreviations: HU, hydroxyurea; CI, confidence interval; df, degrees of freedom.

## Data Availability

The data presented in this study are available upon request from the corresponding author.

## References

[1] Alhawiti N.M., Shubair M.M., Alharthy A., Al-Khateeb B.F., Aldahash R., Aleissa B., Angawi K., AlJumah M., Almutairi A., Almutairi M.N. (2025). The Prevalence and Predictors of Sickle Cell Anemia in the Saudi Arabia General Population: Findings from a Cross-Sectional Study. Healthcare.

[2] Kayle M., Blewer A.L., Pan W., A Rothman J., Polick C.S., Rivenbark J., Fisher E., Reyes C., Strouse J.J., Weeks S. (2024). Birth Prevalence of Sickle Cell Disease and County-Level Social Vulnerability—Sickle Cell Data Collection Program, 11 States, 2016–2020. MMWR Morb. Mortal. Wkly. Rep..

[3] GBD 2021 Sickle Cell Disease Collaborators (2023). Global, regional, and national prevalence and mortality burden of sickle cell disease, 2000–2021: A systematic analysis from the Global Burden of Disease Study 2021. Lancet Haematol..

[4] Beutler E. (2001). Discrepancies between genotype and phenotype in hematology: An important frontier. Blood.

[5] Sundd P., Gladwin M.T., Novelli E.M. (2019). Pathophysiology of Sickle Cell Disease. Annu. Rev. Pathol..

[6] Xu J.Z., Thein S.L. (2022). Revisiting anemia in sickle cell disease and finding the balance with therapeutic approaches. Blood.

[7] Casale M., Benemei S., Gallucci C., Graziadei G., Ferrero G.B. (2025). The phenotypes of sickle cell disease: Strategies to aid the identification of undiagnosed patients in the Italian landscape. Ital. J. Pediatr..

[8] Ata F., Rahhal A., Malkawi L., Iqbal P., Khamees I., Alhiyari M., Yousaf Z., Qasim H., Alshurafa A., Sardar S. (2023). Genotypic and Phenotypic Composition of Sickle Cell Disease in the Arab Population—A Systematic Review. Pharmgenomics Pers. Med..

[9] Hudnall M., Reed-Maldonado A.B., Lue T.F. (2017). Advances in the understanding of priapism. Transl. Androl. Urol..

[10] Anele U.A., Le B.V., Resar L.M.S., Burnett A.L. (2015). How I treat priapism. Blood.

[11] De Niro A.J.N., Duarsa G.W.K., Yudiana I.W., Prayudi N.G., Tirtayasa P.M.W., Santosa K.B., Kloping Y.P. (2023). Predictors of priapism incidence and recurrence in sickle cell disease patients. Afr. J. Urol..

[12] Idris I.M., Burnett A.L., DeBaun M.R. (2022). Epidemiology and treatment of priapism in sickle cell disease. Hematol. Am. Soc. Hematol. Educ. Program.

[13] Whyte N., Morrison-Blidgen B., Asnani M. (2021). Priapism in Sickle Cell Disease: An Evaluation of the Knowledge of an at Risk Population in Jamaica. Sex. Med..

[14] Duarte R.L.M., Magalhães-da-Silveira F.J., Gozal D. (2023). Screening for obstructive sleep apnea: Comparing the American Academy of Sleep Medicine proposed criteria with the STOP-Bang, NoSAS, and GOAL instruments. J. Clin. Sleep Med..

[15] Alghalyini B., Zaidi A.R.Z., Atif K., Mosharraf N., Tamim H., Qureshi M.N. (2024). Priapism Presentations in a Saudi Arabian Emergency Department: A Retrospective Study at a Tertiary Care Hospital. Healthcare.

[16] Al-Awamy B., Taha S.A., Naeem M.A. (1985). Priapism in association with sickle cell anemia in Saudi Arabia. Acta Haematol..

[17] Alsultan A., Alabdulaali M.K., Griffin P.J., AlSuliman A.M., Ghabbour H.A., Sebastiani P., Albuali W.H., Al-Ali A.K., Chui D.H.K., Steinberg M.H. (2014). Sickle cell disease in Saudi Arabia: The phenotype in adults with the Arab-Indian haplotype is not benign. Br. J. Haematol..

[18] Jastaniah W. (2011). Epidemiology of sickle cell disease in Saudi Arabia. Ann. Saudi Med..

[19] Idris I.M., Yusuf A.A., Ismail I.I., Borodo A.M., Hikima M.S., Kana S.A., Aliyu T., Musangedu K., Jibrilla A.U., Aji S.A. (2025). A controlled trial for preventing priapism in sickle cell anemia: Hydroxyurea plus placebo vs hydroxyurea plus tadalafil. Blood.

[20] van Driel M.F. (2015). Physiology of Penile Erection-A Brief History of the Scientific Understanding up till the Eighties of the 20th Century. Sex. Med..

[21] Silveira T.H.R., Calmasini F.B., de Oliveira M.G., Costa F.F., Silva F.H. (2024). Targeting heme in sickle cell disease: New perspectives on priapism treatment. Front. Physiol..

[22] Hotta Y., Kataoka T., Kimura K. (2019). Testosterone Deficiency and Endothelial Dysfunction: Nitric Oxide, Asymmetric Dimethylarginine, and Endothelial Progenitor Cells. Sex. Med. Rev..

[23] Miller D., Gurayah A., Weber A., Schuppe K., Zarli M., Dullea A., Hwang K., Ramasamy R. (2023). Seasonal Variation in Serum Testosterone Levels: Evidence from 2 Large Institutional Databases. Urol. Res. Pract..

[24] Ördek E., Görür S., Gökalp F., Kuru D., Uçurmak F. (2025). The Management of Ischemic Priapism Due to Sickle Cell Disease and Other Etiologies: Treatment Strategies and Indications for Penile Prosthesis Implantation in an Endemic Region. Medicina.

[25] Tan B.K.J., Teo Y.H., Tan N.K.W., Yap D.W.T., Sundar R., Lee C.H., See A., Toh S.T. (2022). Association of obstructive sleep apnea and nocturnal hypoxemia with all-cancer incidence and mortality: A systematic review and meta-analysis. J. Clin. Sleep Med..

[26] Alsalman M., Alkhalifa H., Alkhalifah A., Alsubie M., AlMurayhil N., Althafar A., Albarqi M., Alnaim A., Khan A.S. (2021). Hydroxyurea usage awareness among patients with sickle-cell disease in Saudi Arabia. Health Sci. Rep..

[27] Mejias-Figueroa J.A., Saraf S.L., Takemoto C.M., Rodeghier M., DeBaun M.R. (2025). Priapism before and after hematopoietic stem cell therapy in individuals with sickle cell disease. Blood Adv..

[28] Davis B.A., Allard S., Qureshi A., Porter J.B., Pancham S., Win N., Cho G., Ryan K., Haematology T.B.S.F. (2017). Guidelines on red cell transfusion in sickle cell disease Part II: Indications for transfusion. Br. J. Haematol..

[29] Badewa A.A., Fidelis W., Burnett A.L. (2025). Hydroxyurea in the management of sickle cell disease-associated priapism: A scoping review. Int. J. Impot. Res..

[30] Idris I.M., Abba A., Galadanci J.A., Aji S.A., Jibrilla A.U., Rodeghier M., Kassim A., Burnett A.L., DeBaun M.R. (2022). Incidence and predictors of priapism events in sickle cell anemia: A diary-based analysis. Blood Adv..

